# Pflegende Angehörige in der hausärztlichen Praxis – Hausärztliche Sichtweisen und Lösungsansätze

**DOI:** 10.1007/s00391-021-02000-8

**Published:** 2021-12-21

**Authors:** Yvonne Marx, Thomas Frese, Alexander Bauer

**Affiliations:** Medizinische Fakultät, Institut für Allgemeinmedizin, Martin-Luther Universität Halle-Wittenberg, Magdeburger Str. 8, 06112 Halle (Saale), Deutschland

**Keywords:** Angehörigenpflege, Allgemeinmedizin, Identifikation, Assessment, Betreuung, Caring relatives, General practitioner, Identification, Assessment, Support

## Abstract

**Hintergrund:**

Frühes Erkennen gesundheitlicher Belastungen pflegender Angehöriger sowie die Koordination adäquater Maßnahmen obliegen zumeist dem Hausarzt. Dessen individuelle Perspektiven und Ansätze zu Identifizierung und Unterstützung pflegender Angehöriger wurden bisher kaum untersucht.

**Ziel der Arbeit:**

Die Studie untersucht daher subjektive Sichtweisen von Hausärzten im Hinblick auf Identifikation und Betreuung pflegender Angehöriger in der hausärztlichen Praxis. Mithilfe der Ergebnisse sollen Erleichterungsbedarfe und Bedingungen für eine Implementierung eines Instruments zur Identifikation in der Praxis identifiziert werden.

**Material und Methoden:**

Zur Beantwortung der Fragestellung wurden 12 leitfadengestützte Experteninterviews mit niedergelassenen Hausärzten aus Sachsen-Anhalt durchgeführt. Für die Auswertung der Interviews wurde das Verfahren der qualitativen Inhaltsanalyse nach Mayring angewendet.

**Ergebnisse:**

Die Auseinandersetzung mit subjektiven Sichtweisen bestätigt die grundlegende zentrale Stellung von Hausärzten bezüglich der Betreuung pflegender Angehöriger. Sie zeigt aber auch Ansatzpunkte mit Potenzial zu Verbesserung bzw. Erleichterung der Versorgung pflegender Angehöriger: Zeitliche Probleme und mangelnde Kommunikation erschweren die Betreuung. Ambulante Pflegedienste und regionale (Beratungs‑)Angebote sind vorhanden; eine systematische Zusammenarbeit und Koordination fehlt.

**Diskussion:**

Ein Screeninginstrument für pflegende Angehörige (Identifikation, Messen der empfundenen Belastung) stellt eine sinnvolle Intervention dar. Die besonderen Anforderungen und Eigenschaften von Interventionen in der allgemeinmedizinischen Praxis müssen systematisch erforscht werden und die Häusliche Pflege-Skala entsprechend angepasst und implementiert werden. Die Zusammenarbeit aller Akteure nimmt zukünftig eine zentrale Stellung ein.

**Zusatzmaterial online:**

Zusätzliche Informationen sind in der Online-Version dieses Artikels (10.1007/s00391-021-02000-8) enthalten.

## Abstract (en)

**Background:**

General practitioners are usually responsible for the early detection of burden due to informal care and the coordination of adequate measures. Their individual perspectives and approaches for identifying and supporting caregiving relatives have hardly been examined yet.

**Objective:**

The study therefore examined subjective perspectives of general practitioners regarding the identification and care of family caregivers in general practitionersʼ practices. The results will help to evaluate the needs for improvement and conditions for implementation of an instrument to identify informal carers.

**Material and methods:**

To answer the question, we conducted 12 guideline-based expert interviews with general practitioners from Saxony-Anhalt. To evaluate the interviews, we used qualitative content analysis (Mayring).

**Results:**

With respect to the medical care and support of caring relatives, the examination of subjective attitudes and perspectives confirmed that general practitioners occupy a central position. It also showed possibilities to improve or to facilitate support of caring relatives. Time-dependent problems and a lack of communication complicate support. Home care services and regional consulting services are available but a systematic cooperation and coordination of competences is still lacking.

**Conclusion:**

A screening instrument for family caregivers (identification, measuring subjective burden) represents a meaningful intervention. The special requirements and characteristics of interventions used in general medical practice have to be researched, the burden scale for family caregivers should be adapted and implemented accordingly. The cooperation of all actors must be a central aspect of future efforts.

In vielen Fällen können ein Verbleib und eine umfassende Betreuung pflegebedürftiger Personen im häuslichen Bereich nur durch pflegende Angehörige (pA) sichergestellt werden. Frühes Erkennen gesundheitlicher Belastungen durch informelle Pflege sowie die Koordination adäquater Maßnahmen obliegen zumeist dem Hausarzt. Deren Einstellungen und individuelle Sichtweisen sind daher von besonderer Relevanz, um z. B. den Bedarf nach Interventionen abzuschätzen und die (Neu/Weiter‑)Entwicklung eines Instruments zur frühen Identifikation und Erfassung der Pflegebelastung zu ermöglichen.

## Hintergrund und Fragestellung

Pflegende Angehörige übernehmen den mit Abstand größten Anteil an längerfristiger Pflege ihrer zumeist älteren und chronisch kranken Verwandten oder Freunde. Damit verbunden ist ein höheres Risiko für Überforderung der Pflegepersonen durch die anhaltend hohe zeitliche, finanzielle und emotionale Belastung [z. B. 4, 22].

Die oftmals unspezifischen Auswirkungen anhaltender psychischer und körperlicher Belastungen pflegender Angehöriger frühzeitig zu erkennen, ist ein Schlüsselfaktor für eine angemessene Koordination ggf. vorhandener Ressourcen und Vermittlung individueller Hilfen zum Erhalt einer funktionalen Pflegebeziehung ohne gravierende chronische Überlastung. Hausärzte (HÄ) haben eine zentrale Rolle beim Erkennen von Belastungen und als Lotse für Patienten und ihre pA [5, 21, 22].

Jedoch sprechen pA gesundheitliche Probleme durch Pflege selten von allein an und fordern noch seltener proaktiv Unterstützung ein [5, 22, 28]. Auch Angehörige der Gesundheitsberufe und Sozialdienste bieten angemessene Unterstützung von sich aus in vielen Fällen zu selten an [4, 5, 15, 22, 28]. Die S3-Leitlinie „Pflegende Angehörige von Erwachsenen“ (2018) empfiehlt daher (wiederholte) Assessments der Pflegesituation und Belastung z. B. bei Übernahme einer Pflegetätigkeit oder gesundheitlichen Beschwerden der Angehörigen [6]. Die in der Leitlinie enthaltene Kurzversion der Häuslichen Pflege-Skala (HPS, 10 Items) wird empfohlen, um die individuellen Belastungen der pA zu erfassen und auch im Verlauf zu beurteilen [10, 11].

Individuelle Sichtweisen der HÄ zur Identifizierung und Unterstützung pflegender Angehöriger wurden bisher kaum untersucht [15]. Einen Aufschlag machte Schneemilch (2018). Sie befragte 10 Hausärzte zu ihrer Beziehung zu pA und stellte heraus, dass die Intensität, mit der Hausärzte pA betreuen, stark variiert und eine große Diskrepanz zwischen benötigter und zur Verfügung stehender (Gesprächs‑)Zeit eine adäquate Betreuung erheblich erschwert. Des Weiteren behindern oftmals Kommunikations- und Informationsdefizite der involvierten Personen (z. B. nichtangesprochene Probleme usw.) sowie eine inadäquate Vergütung der Gespräche die zeitnahe Versorgung [23].

Ziel der Untersuchung ist, Potenziale zur frühen Erkennung und zur besseren Implementierung in den Praxisalltag zu identifizieren.

## Methode

Die beschriebene Fragestellung wurde qualitativ durch leitfadengestützte Experteninterviews bearbeitet. Zwischen November 2019 und Juli 2020 wurden 12 Interviews mit HÄ in Sachsen-Anhalt durchgeführt. Für die Auswertung der Interviews wurde das Verfahren der qualitativen Inhaltsanalyse nach Mayring angewendet [14, 19]. Erläuterungen zum Studiendesign und die demografischen Daten der Teilnehmer (Tab. 1) sind online verfügbar (Zusatzmaterial online: Supplement 1).

## Ergebnisse

Die Ergebnisse sind nachfolgend für die einzelnen Fragenschwerpunkte dargestellt.

### Identifikation: Symptome und Verhalten bei Verdacht

Symptome, die einen Verdacht auf pflegebedingte Überlastung auslösen, sind mannigfaltig. Eine langjährige Arzt-Patient-Beziehung sowie regelmäßige Hausbesuche begünstigen schnelles Erkennen. Symptome, die in den Interviews als hinweisend genannt wurden, sind in Tab. 1 zusammengefasst.

**Table 1:** 

„Klassische“ Konstellation	Patientenberichtete Symptome	Messbare Symptome	Mimik/Gestik/Verhalten
Ältere Ehepartner (70+) (oft: Frau pflegt Mann)	Häufige Kopfschmerzen	Bluthochdruck	„Vorgeschobene“ Anlässe: Probleme auf der Arbeit, in der Beziehung
Tochter (50+) pflegt Eltern	Häufige Rückenschmerzen (akut/verstärkt)	Herz-Rhythmus-Störungen	Trauriger, erschöpfter Ausdruck
	Depressive Symptome		Aufstöhnen, Seufzen, Weinen
	Erschöpfung		Hängende Schultern, gebückte Haltung
	Schlafstörungen		Stimmungslabilität
	Bauchschmerzen		Aggressiver Ton
	Magenbeschwerden		Veränderung des „bekannten“ Auftretens (Ernährungszustand ↓, Hygiene ↓)
	Somatisierung: viele (untypische) Symptome ohne erklärbare organische Ursache		

Eine Arztkonsultation erfolgt i. d. R. nicht allein, sondern zusammen mit dem zu Pflegenden. Dann hat dieser Priorität. Wird der pA allein vorstellig, geschieht dies zumeist aus einem anderen Anlass – pflegebedingte Probleme werden nicht angesprochen. In einigen Fällen ist dem Hausarzt bekannt, dass sein Patient Angehörige pflegt. Dies sind oft „klassische“ Pflegesituationen.

*(…) die sagen nicht, ich pflege übrigens meine Mutter. Das muss der Arzt konkret selbst ansprechen. Was eigentlich belastet, stellen die hinten an. Die kommen meist zusammen rein, und dann wollen sie die nicht noch zusätzlich belasten. (B2_Abs. 34)*

Alle in ländlichen Regionen tätigen Befragten (*n* = 7) gaben an, familiäre Verhältnisse und Pflegesituationen in den meisten Fällen zu kennen. Auch Familienmitglieder sind oft langjährige Patienten in der Praxis. Dann spricht der Arzt sie, wenn möglich, bereits zu Beginn des Pflegegeschehens oder im vertrauten Umfeld bei Hausbesuchen an. Insbesondere in ländlichen Regionen tätige Hausärzte nennen Hausbesuche als wichtiges Betreuungsinstrument.

*Ja, das spreche ich aktiv an. Dann wird schon im Erstgespräch drauf hingewiesen, dass die Angehörigen sich nicht vergessen dürfen, auch Hilfen in Anspruch nehmen können (…). Und dann haben Sie erst mal ihren Fuß in der Wohnung drin, im wahrsten Sinne des Wortes und dann geht das weiter. (B4_Abs. 50)*

Auf pflegebedingte Belastung geschlossen wird oft bei nicht zusammenpassenden (psycho-)somatischen Beschwerden ohne organische Ursache, die von hausärztlicher Seite nicht zugeordnet werden können. Insbesondere von länger tätigen HÄ wird Aspekten der Intuition und der Wahrnehmung dieser Symptome eine große Bedeutung zugesprochen.

*(…) das sind typischerweise Symptome, die man nicht zuordnen kann. Also ohne organisches Korrelat, wenn einer mit vier verschiedenen Organsystemen kommt, wo er Probleme hat, dann ist es sehr verdächtig (…). (B8_Abs. 19)*

Alle Interviewpartner sprechen pflegebedingte Belastung bei Verdacht aktiv an. Zeitliche Aspekte spielen bei der Erkennung eine entscheidende Rolle, evtl. wird ein Gespräch verschoben und findet zeitlich verzögert statt.

*Also, wenn man es einfach mal verschweigt, zwar schon sieht, da könnte ja vielleicht ’n Problem liegen, ist das etwas einfacher als zu sagen, jetzt erzählen Sie mal. Das kostet sehr viel Zeit und da kann man sich so ’ne Sprechstunde auch sprengen. Da kann man auch mal ’ne dreiviertel Stunde mit dem Angehörigen zubringen, nur mit reinem Gespräch. Das kann man nicht morgens um acht am Montag machen, dann kann man die anderen gleich nach Hause schicken *(lacht). (B2_ Abs. 26)

### Bewusstsein für Belastungen der pflegenden Angehörigen

Das Bewusstsein für die durch Pflege resultierende Überforderung und Selbstaufgabe der pA ist bei allen HÄ vorhanden. Die Belastungen der pA werden von ihnen als hoch bis kritisch eingeschätzt.

*Ich habe regelmäßig das Gefühl, dass ich die Angehörigen davor bewahren muss, sich selbst zu überfordern. (B7_ Abs. 3)*

*(…) die eben finanziell nicht so gut dastehen und dann wirklich als alleinige Bezugsperson einen Kranken pflegen, das halte ich für fast nicht umsetzbar, weil derjenige gibt sich dann in dem Moment meiner Meinung nach auf. (B9_ Abs. 23)*

### Bedeutung pflegender Angehöriger in der hausärztlichen Praxis

Pflegenden Angehörigen werden innerhalb des Pflegegeschehens unterschiedliche, aber zumeist essenzielle Rollen zugeschrieben. Ausführungen zu den zugeordneten Rollen und Beispiele sind online einzusehen (Zusatzmaterial online: Tab. 3, Kategoriensystem zur Ausprägung „Bedeutung der pA“).

### Betreuungsaufwand und -probleme

Die Mehrzahl der interviewten HÄ gibt an, dass Gespräche und Koordination von Hilfsmaßnahmen sehr zeitaufwendig sind (*n* = 10).

*Es fordert Zeit, die Koordination von externen Hilfsmöglichkeiten, das Hilfebedürfnis der Angehörigen (…), also es ist ein aufwendiges Gesprächsthema. Das sind keine Fünf-Minuten-Termine. (B7_ Abs. 13)*

Möglichkeiten zur Entlastung der pA sind unseren Interviewpartnern zufolge oft vorhanden und werden angeboten. Pflegende Angehörige nehmen diese aber teilweise bewusst nicht in Anspruch, da sie oder die zu pflegende Person z. B. fremde Hilfen oder ein fremdes Umfeld ablehnen. Hausärzte beschreiben diesen Umstand als frustrierend. In Konstellationen, in denen Kinder Eltern pflegen, behindern oft Autoritätsprobleme die Einhaltung der Maßnahmen. Ebenso problematisch sind mangelnde Adhärenz zu hausärztlichen Empfehlungen und (vermeintlich gut gemeinte) eigenmächtige Handlungen der Pflegenden.

*(…) wir hatten ’ne Patientin, da hat’s der Mann und die Tochter nicht geschafft, die Mutti zu lagern. Hat sich wund gelagert, Druckentlastung ist in erster Linie das A und O. Aber das wollen’se nicht hören. Na, wie sollen wir’s denn dann machen? (…) Müssen wir ’se vielleicht mal für 14 Tage oder vier Wochen in ’ne stationäre Einrichtung geben, dann wird abgeblockt. (B6_Abs. 29)*

### Rollenverständnis der Hausärzte

Die interviewten HÄ fühlen sich bezüglich der Betreuung pflegender Angehöriger vollumfänglich zuständig. Der betreuende Hausarzt hat in dieser Rolle eine ganzheitliche Sicht auf die Patienten, deren familiäre Umstände sowie die Erfahrung, vorliegende Sachverhalte zu analysieren und entsprechend zu koordinieren. Wenn möglich, wird an medizinische Fachangestellte mit entsprechender Zusatzausbildung delegiert (VERAH). Diese Möglichkeit wird als große Hilfe empfunden.

*(…) und somit ist dann der Hausarzt genau das, nämlich der zentrale Gatekeeper ist ja sogar untertrieben, es ist ja sogar der Allesmacher, egal welches Problem, das meiste löst irgendwie der Hausarzt. (B1_Abs. 28)*

### S3-Leitline „Pflegende Angehörige“ und Häusliche Pflege-Skala

Ein Assessment für pA gibt es bisher nicht. Die S3-Leitlinie „Pflegende Angehörige“ soll eine Hilfe darstellen, um eine frühe Erkennung, Diagnostik und Koordination angemessener Maßnahmen zu ermöglichen. Zwei der 12 befragten HÄ war sie bekannt, wurde aber nicht genutzt. Nach Angaben der Interviewpartner werden alternativ in praxisinternen Fragebogen teilweise Pflegeaktivitäten erfragt (*n* = 3), die in der Leitlinie aufgeführte Häusliche Pflege-Skala (HPS) aber nicht verwendet. Ein spezielles Instrument wird nur von 2 HÄ gewünscht.

*Es gibt ganz viele Screenings. Und genau sowas könnte man auch für pA anbieten. Da scheuen viele zurück, das ist dann sehr aufwendig, weil man muss die richtige Ziffer bei dem richtigen Patienten mit der richtigen Diagnose abrechnen. Ich glaube, dass so’n Screening für pA sinnvoll wäre und denen auch helfen würde. Nicht jedes Screening hat ja Sinn, aber hier könnte man auch durchaus Defizite rausfiltern. (B2_Abs. 28)*

Der Bedarf nach einem weiteren Assessment ist vor einer bereits existierenden hohen Anzahl an Assessments und Instrumenten für verschiedene Krankheitsbilder für die befragten HÄ zunächst nicht ausdrücklich gegeben (*n* = 6).

*Schwierig … wir haben genug Assessments in der Pflege. (…) Aber zu den pA, da noch’n Instrument … vor allen Dingen muss man ja dann aus ’nem Assessment auch ’ne Folge ziehen. (B4_Abs. 82)*

## Diskussion

Die Studie bestätigt die zentrale Stellung von HÄ bezüglich der Erkennung, Beratung und Betreuung pflegender Angehöriger. Sie zeigt Ansatzpunkte zur Verbesserung der Versorgung in der allgemeinmedizinischen Praxis, aber auch kontroverse Sichtweisen von HÄ auf.

Dass ein proaktives Ansprechen einer Pflegebelastung nicht systematisch erfolgt, macht deutlich, dass mangelnde zeitliche Ressourcen und längere Gespräche einen geplanten Praxisablauf erschweren. Als zeitliche Spanne wurde dafür von den interviewten Ärzten ein mindestens 10-minütiges Gespräch angegeben. Tatsächlich beträgt die durchschnittliche Gesprächszeit pro Patient in Deutschland max. 7 min [7]. Insbesondere psychosomatische Symptome erwecken den Verdacht auf Überlastung durch Pflege. Ein Ansprechen dieser komplexen Probleme macht auf zeitlicher Ebene Einschränkungen im weiteren Praxisablauf notwendig [16].

Die Aussagen zur S3-Leitline lassen ähnlich wie bei Schneemilch (2018) u. a. Rückschlüsse auf Verbesserungspotenzial hinsichtlich der Implementierung der Leitlinie zu. Von einer generellen Leitlinienmüdigkeit kann im Hinblick auf die Ergebnisse anderer Studien zur Akzeptanz und Nutzung von Leitlinien in der hausärztlichen Praxis nicht ausgegangen werden [1, 20]. Leitlinien mit überwiegend pharmakotherapeutischen Inhalten erfahren eine höhere Akzeptanz als z. B. Leitlinien mit eher kommunikativen Inhalten, deren Umsetzung im Wesentlichen von zeitlichen und strukturellen Möglichkeiten des Hausarztes abhängt. Die Leitlinie „Pflegende Angehörige“ enthält überwiegend Empfehlungen, die kommunikative und zeitliche Ressourcen der Ärzte und auch proaktives Vorgehen der Patienten erfordern. Möglicherweise führt dieser Umstand zu Umsetzungsproblemen [1, 24].

Wangler et al. (2019) untersuchten in einer deskriptiven Studie die Erwartungen pflegender Angehöriger und deren tatsächlich erlebte Betreuung in der hausärztlichen Praxis. Dazu wurden in Internetforen 204 pA befragt. Die aus der Studie hervorgegangenen Problempunkte korrelieren mit Aussagen der befragten Ärzte, die sich als erster Ansprechpartner für pA sehen und mit deren komplexen Situation zumeist vertraut sind. Mangelnde zeitliche Ressourcen und unzureichende aktive Ansprache der pA als Pflegeperson wurden ebenfalls als problematisch identifiziert [26]. Dieser Umstand könnte u. a. auch auf die beschriebene unzureichende Nutzung eines Screeninginstruments zurückzuführen sein, obwohl sowohl im nationalen als auch im internationalen Kontext standardisierte Instrumente zu Erkennung und Erfassung einer pflegebedingten Belastung existieren (z. B. Häusliche Pflege-Skala – HPS; engl.: Burden Scale for Family caregivers – BSFC).

Ein Instrument zur frühen Erkennung und Verlaufsbeurteilung, das strukturelle Barrieren und knappe zeitliche Ressourcen berücksichtigt, kann eine adäquate Lösung darstellen. Bei Assessments und Interventionen ist es schwer, den Implementierungserfolg vorherzusagen, da weitere Faktoren in der (haus)ärztlichen Praxis diesen beeinflussen [12]. Deshalb halten es die Autoren für notwendig, die besonderen Anforderungen und Eigenschaften von Interventionen in der allgemeinmedizinischen Praxis systematisch zu erforschen (z. B. syst. Review, Fokusgruppen – Diskussion), um ein adäquates Instrument zu entwickeln oder die HPS entsprechend zu modifizieren.

Die Ausführungen der befragten Ärzte zeigen eine teilweise praktizierte, aber zeitlich schwer realisierbare „Allzuständigkeit“, die sich aufgrund eines empfundenen Fehlens eindeutig definierter Zuständigkeiten und zentraler Koordination auch für (nichtärztliche) Leistungen ergibt, die z. B. im SGB XI geregelt sind (z. B. § 7a SGB XI – individuelle Pflegeberatung) [8, 25].

Um eine umfassende Betreuung pflegender Angehöriger gewährleisten zu können, sind die Vernetzung und Zusammenarbeit mit lokalen Akteuren essenziell [27]. Mit dem Pflege-Weiterentwicklungsgesetz 2008 strebten Sachsen-Anhalt und Sachsen die „vernetzte Pflegeberatung“ an. Bestehende Strukturen sollten ausgebaut und vernetzt werden. Notwendige Voraussetzungen sind regional vorhanden (ambulante Pflegedienste, Beratungsstellen) und werden auch genutzt. Sowohl vorhergegangene Evaluationen als auch unsere Ergebnisse zeigen jedoch, dass eine aktive Kooperation und Kommunikation aller beteiligten Akteure besonders an der Schnittstelle Pflege/Pflegeberatung – Hausarztpraxis notwendig ist, aber bisher als nicht umgesetzt betrachtet werden kann [2, 27]:

Eine häufige Zusammenarbeit zwischen Pflegestützpunkten (PSP) und HÄ erfolgt laut einer aktuellen Erhebung in Brandenburg (*n* = 41) von Hahnel et al. nur bei 14,6 % der PSP [13]. Zu einem ähnlichen Fazit kommen Braeske et al. (2018) in einer bundesweiten Online-Befragung mit 148 Pflegestützpunkten. Als ausdrücklich „noch nicht befriedigend“ wird neben der Zusammenarbeit mit Krankenhäusern und Fachkliniken (28 %), niedergelassenen (Fach‑)Ärzten (35 %) von 40 % der befragten PSP die Zusammenarbeit mit HÄ bezeichnet [3].

Durch unzureichende Kooperation und Kommunikation kommt es häufig zu vermeidbaren Versorgungsbrüchen bei der zu pflegenden Person und deren pA. Neben der Entwicklung eines adäquaten Assessments zur frühen Identifikation muss die Zusammenarbeit zwischen Beratungsstellen und HÄ verbessert werden.

Das Konzept FIDEM Niedersachsen als gemeinsames Netzwerk von Arztpraxen und nichtärztlichen Unterstützungsangeboten für Menschen mit einer Demenzerkrankung stellt einen vielversprechenden Ansatz dar. Die Netzwerkteilnehmenden sind in der Regel Anbieter von Pflegeberatung, niedrigschwelligen Betreuungs- und Entlastungsangeboten, Ergotherapiepraxen (ggf. mit entsprechender Weiterbildung) und Selbsthilfegruppen für Angehörige und zu Pflegende. Pflegende und ihre pA werden in einem Patientengespräch beim Hausarzt über die Möglichkeiten des Netzwerks informiert. Dieser darf (bei Einwilligung und nach Entbindung der Schweigepflicht) deren Kontaktdaten z. B. an eine ausgewählte Pflegeberatung weiterleiten (strukturiertes Faxformular). Die aufsuchende Arbeitsweise der nichtärztlichen Akteure ist ebenso zentrales Merkmal dieses Konzeptes wie eine Rückmeldung dieser an die Hausarztpraxis über erfolgte Kontaktaufnahme sowie weitere Entwicklungen. Anbieter nichtärztlicher Leistungen vermitteln die Patienten und ihre pA bei Bedarf untereinander weiter. Die regionale Koordination und Umsetzung des Konzeptes übernehmen (Senioren- und) Pflegestützpunkte in der Wahrnehmung ihrer Funktion des Care-Managements [18]. Die Wirksamkeit eines neu entwickelten Assessments zur frühen Identifikation pflegender Angehöriger kann innerhalb des Netzwerks getestet werden.

## Limitationen

Den Autoren ist bewusst, dass die teilweise gesteuerte Auswahl der Studienteilnehmer nicht zur Rekrutierung einer repräsentativen Stichprobe führen kann. Vorteilig war jedoch, dass so die spezifische Fragestellung mit einer festgelegten Expertengruppe unter Einbezug vieler Faktoren (Alter, Praxissitz etc.) bearbeitet werden konnte [9].

Durch Anwesenheit des Interviewers in einem persönlichen Interview besteht die Gefahr durch Verzerrungen durch sozial erwünschtes Antwortverhalten [17]. So wird eine unzureichende Vergütung längerer Gespräche als unproblematisch benannt. Zu vermuten ist allerdings, dass einige normativ tabuisierte Angaben wie ökonomische Überlegungen, die im persönlichen Interview scheinbar eine untergeordnete Rolle spielen, tatsächlich mehr Einfluss haben.

## Fazit für die Praxis

- Durch eine klare Definition und Abgrenzung der Zuständigkeiten der verschiedenen Akteure (z. B. Hausärzte, Pflegekassen etc.) können Versorgungsbrüche minimiert und die Betreuung pflegender Angehöriger (pA) erleichtert werden.
- Die bisher nicht erreichte Vernetzung erfordert eine systematische Verschränkung von Case- und Care-Management mit einer guten Koordination und Kooperation der bestehenden Akteure (inkl. Hausärzte).
- Ein schnell verfügbarer Überblick über regionale Strukturen und Organisationen erleichtert z. B. die Vermittlung von Entlastungsangeboten.
- Pflegende Angehörige sollten sensibilisiert werden, sich selbst als (belastete) Pflegepersonen anzusehen und resultierende Probleme beim Hausarzt aktiv anzusprechen.

## Supplementary Information





