# Supplementary material for: Caring relatives in General practice—General practitionersʼ perspectives and solution approaches
Source: Z Gerontol Geriatr. 2021 Dec 21;56(1):23–8. [Article in German] doi: 10.1007/s00391-021-02000-8 (PMC9877090; doi:10.1007/s00391-021-02000-8)
Supplement: Supplementary file 1 [file 391_2021_2000_MOESM1_ESM.docx]

**Supplement 1: Studiendesign und Methode**

Leitfadenerstellung

Ein Leitfaden für die Experteninterviews wurde auf Grundlage des aktuellen Kenntnisstandes und der zentralen Fragestellungen entwickelt. Er enthält Fragen zur Bedeutung pflegender Angehöriger in der hausärztlichen Praxis, zum tatsächlichen und empfundenen Aufwand sowie zu Problemen in Bezug auf die Betreuung pflegender Angehöriger. Des Weiteren wurden die Teilnehmer gebeten, ihre Sichtweise der eigenen Rolle als AM zu umreißen. Das individuelle Verhalten bei einem Verdacht auf pflegebedingte Belastungen sowie Symptome, die erfahrungsgemäß aufhorchen lassen, wurden ebenso erfragt. Der Leitfaden wurde im Vorfeld mit drei Hausärzten getestet und entsprechend deren Anmerkungen adaptiert. Diese Pre-Test-Interviews wurden nicht verwendet, da aufgrund vorbesprochener Details Verzerrungen oder Beeinflussung vermutetet werden mussten. Ergänzend wurde ein Kurzfragebogen erstellt, der u. a. Angaben zum Alter, zur Dauer der Niederlassung, Region (städtisch/ländlich) sowie zu Zusatzqualifikationen des Praxispersonals enthielt.

Rekrutierung und Sample

Die Adressen der niedergelassenen AM wurden dem Portal der Kassenärztlichen Vereinigung Sachsen-Anhalt entnommen (insgesamt n=1160), E-Mail-Adressen wurden ebenfalls im Internet recherchiert. Zunächst wurden jeweils 50 AM zufällig ausgewählt und postalisch (Anschreiben, Teilnehmerinformation sowie ein frankierter Rückumschlag) mit der Bitte um Teilnahme kontaktiert. Weitere 50 erhielten diese Information und Anfrage per E-Mail. Ausschlusskriterien wurden bis auf eine Nichteinwilligung zur Teilnahme nicht definiert. Im späteren Verlauf wurden drei AM gezielt nachrekrutiert, um die gewünschte Heterogenität (unterschiedliche Altersgruppen, Praxisformen, Regionen) zu erreichen. Alle Teilnehmer wurden vorher über das Vorhaben und Vorgehen in Kenntnis gesetzt und gaben ihr schriftliches Einverständnis. Zwischen 11/2019 und 07/2020 wurden 12 Interviews durchgeführt, bei einem Interview nahm eine angestellte VERAH auf Wunsch des Arztes mit teil. Die Interviews dauerten zwischen 20 bis 45 Minuten. Sie wurden von YM digital aufgezeichnet und wörtlich transkribiert (F4transkript, https://www.audiotranskription.de/f4), persönliche Daten in diesem Arbeitsschritt pseudonymisiert. Die Richtigkeit der Transkripte und vollständige Pseudonymisierung wurde überprüft. Die demographischen Daten der Teilnehmer können Tab. 1 entnommen werden.

Tab. 1 Demographische Daten der Interviewteilnehmer

| TN | Ge-schlecht | Altersgruppe | Dauer NL/AB  in Jahren | Praxisform | PP | davon mit ZA: | Region |
| --- | --- | --- | --- | --- | --- | --- | --- |
| B1 | **♂** | 31-40 | 5 | BAG | 14 | 5 (div.) | städt. |
| B2 | **♀** | 31-40 | 5 | EP | 4 | 1 (V) | städt. |
| B3 | **♀** | ≤ 30 | < 1 | Anstellung | k. A | k. A. | städt. |
| B4 | **♀** | 51-60 | 19 | EP | 2 | 1 (V) | ländl. |
| B5 | **♀** | 41-50 | 12 | EP | 4 | 2 (V) | ländl. |
| B6 | **♂** | > 60 | 36 | MVZ | 40 | 20 (div.) | ländl. |
| B7 | **♂** | 51-60 | 16 | EP | 5 | 1 (V) | ländl. |
| B8 | **♂** | > 60 | k. A. | EP | k. A. | k. A. | ländl. |
| B9 | **♂** | 31-40 | 2,5 | BAG | 5 | 1 (V) | ländl. |
| B10 | **♂** | 41-50 | 17 | BAG | 7 | 2 (V) | städt. |
| B11 | **♂** | 41-50 | 18 | EP | 3 | 1 (V) | ländl. |
| B12 | **♀** | 51-60 | k.A. | EP | 4 | 2 (V) | städt. |

NL/AB: Niederlassung/Arbeitsverhältnis, Praxisformen: BAG – Berufsausübungsgemeinschaft, EP – Einzelpraxis, PP: Praxispersonal, davon mit Zusatzausbildung (ZA), V: VERAH^[[1]](#footnote-1)^

Analyse

Für die Auswertung der Interviews wurde das Verfahren der qualitativen Inhaltsanalyse nach Mayring angewendet [9, 14, 19]. Die Ermittlung der Kategorien erfolgte deduktiv aus den Basishypothesen des Leitfadens und (ergänzend) induktiv aus der Aufarbeitung des Interviewmaterials [19]. Zur Aufarbeitung der Daten wurde das Programm F4analize genutzt (https://www.audiotranskription.de/f4-analyse).

1. Versorgungsassistentin in der Hausarztpraxis [↑](#footnote-ref-1)
