# Supplementary material for: Caring relatives in General practice—General practitionersʼ perspectives and solution approaches
Source: Z Gerontol Geriatr. 2021 Dec 21;56(1):23–8. [Article in German] doi: 10.1007/s00391-021-02000-8 (PMC9877090; doi:10.1007/s00391-021-02000-8)
Supplement: Supplementary file 2 [file 391_2021_2000_MOESM2_ESM.docx]

Supplement 2: Bedeutung pflegender Angehöriger in der hausärztlichen Praxis

PA werden innerhalb des Pflegegeschehens unterschiedliche, aber zumeist essentielle Rollen zugeschrieben. Aus Sicht der Befragten kann in den meisten Fällen (n = 10) eine umfassende Betreuung im häuslichen Bereich mit entsprechender Gabe von Medikamenten sowie regelmäßige Arztbesuche nur durch pA sichergestellt werden.

Durch Einbezug junger Ärzte in die Interviews (z. B. Weiterbildungsassistenten) und den Vergleich mit Angaben sehr erfahrener Kollegen kann eine Veränderung im Bewusstsein für die Rolle der pA sowie bzgl. des Wissens um Besonderheiten im Umgang mit pA vermutet werden. Während länger tätige AM pA durchgehend als essentiell zur Sicherstellung einer häuslichen Betreuung ansehen (n = 10), sehen jüngere AM diese zunächst z. B. als Bindeglied zwischen dem Arzt und dem zu Pflegenden oder als Begleitperson (n = 2).

Tab. 3: Kategoriensystem zur Ausprägung „Bedeutung der pA in der hausärztlichen Praxis“

| **Kategorie** | **Kodierregeln (i. A. an [9, 14, 19])** | **Ankerbeispiel** | **n** |
| --- | --- | --- | --- |
| **Gering** | PA fungieren lediglich als Begleitpersonen o. ä., übernehmen nicht zwingend notwendige Tätigkeiten bzw. es erfolgt keine weitere Ausführung. |  | 0 |
| **relevant** | Die Zuschreibung einer mäßigen Bedeutung erfolgt, wenn a) pA als mehr als nur Begleitpersonen angesehen werden, aber noch keine Zuschreibung essentieller Rollen und Aufgaben erfolgt und b) keine eindeutige Zuordnung mehr zu gering bzw. noch nicht zu unabdingbar möglich ist | „Die spielen schon eine Rolle, weil die ja auch Bindeglied vor allen Dingen zu den Schwerkranken sind, die fast nur in der Häuslichkeit zu besuchen sind oder die zumindest nicht so mobil sind, um hier regelmäßig in der Praxis aufzuschlagen.“ (B9_ Abs. 3) | 2 |
| **unabdingbar** | Die Zuschreibung einer sehr hohen Bedeutung erfolgt, wenn pA essentielle Aufgaben zugeschrieben werden bzw. ein Verbleib im häuslichen Umfeld oder die Sicherstellung anderer Aktivitäten/der ärztlichen und pflegerischen Versorgung nur durch sie möglich ist. | „Also ich denke immer, dass ohne pA das ganze System zusammenbrechen würde (…) PA sind natürlich nicht nur in Akutsituationen, sondern auch bei einer Langzeitbetreuung unheimlich wichtig. Die können natürlich auch viel mehr leisten, weil sie in der Regel ja da wohnen und eben auch gar nicht anders können als helfen.“ (B1_ Abs. 2) | 10 |
|  |  | „Das ist schon `ne extrem wichtige Rolle, weil die pA die Patienten begleiten, dass Sie überhaupt hier her kommen können und bestimmte Behandlungen sicher stellen, dass sie die Medikamente nehmen, dass sie regelmäßig zum Arzt gehen.“ (B2_ Abs. 2) |  |

n Nennungen
